# Supplementary material for: Demonstrating the utility of Instrumented Gait Analysis in the treatment of children with cerebral palsy
Source: PLoS One. 2024 Apr 9;19(4):e0301230. doi: 10.1371/journal.pone.0301230 (PMC11003627; doi:10.1371/journal.pone.0301230)
Supplement: S1 Appendix — (DOCX) [file pone.0301230.s001.docx]

# Appendix 1: Model Features

## Clinical Features

Features for the CLI model are drawn from the standard clinical evaluation that includes patient history and physical examination. The names listed below are codes from our database, but most of them are intelligible.

#### Diagnosis

dxmod, affected

#### Descriptive

age, HEIGHT, WEIGHT, Sex, SIDE

#### Birth and Developmental History

NICU_Weeks, Delivery_Weeks, Ventilator_Weeks, AGE_AT_DIAG, DEV_FIRST_STEP, DEV_WALK

#### Function and Mobility

GMFCS, FAQ, FAQT

#### Prior and Interval Treatment

prior_Adductor_Release, prior_Psoas_Release, prior_Tibial_Derotation_Osteotomy, prior_Foot_and_Ankle_Bone, prior_Gastroc_Soleus_Lengthening, prior_Femoral_Derotation_Osteotomy, prior_Hams_Lengthening, prior_Rectus_Transfer, prior_Foot_and_Ankle_Soft_Tissue, prior_DFEO, prior_Patellar_Advance, prior_Neural_Rhizotomy, interval_Adductor_Release, interval_DFEO, interval_Femoral_Derotation_Osteotomy, interval_Foot_and_Ankle_Bone, interval_Foot_and_Ankle_Soft_Tissue, interval_Gastroc_Soleus_Lengthening, interval_Hams_Lengthening, interval_Neural_Rhizotomy, interval_Patellar_Advance, interval_Psoas_Release, interval_Rectus_Transfer, interval_Tibial_Derotation_Osteotomy

#### Range of Motion and Alignment

HIP_EXT, HIP_ABD_0, POP_ANG_UNI, KNEE_EXT, EXTEN_LAG, ANK_DORS_0, ANK_DORS_90, ANTEVERSION, BIMAL

#### Strength, Spasticity, Static Motor Control

ABDOM_SEL, ABDOM_STR, BACK_EXT_SEL, BACK_EXT_STR, ADDUCTOR_SPAS, ANT_TIB_SEL, ANT_TIB_STR, EXT_HALL_LONG_SEL, EXT_HALL_LONG_STR, FLEX_HALL_LONG_SEL, FLEX_HALL_LONG_STR, HAMSTRING_SPAS, HIP_ABD_SEL, HIP_ABD_STR, HIP_ADD_SEL, HIP_ADD_STR, HIP_EXT_KN90_SEL, HIP_EXT_KN90_STR, HIP_EXT_SEL, HIP_EXT_STR, HIP_FLEX_SEL, HIP_FLEX_SPAS, HIP_FLEX_STR, KNEE_EXT_SEL, KNEE_EXT_STR, KNEE_FLEX_SEL, KNEE_FLEX_STR, PERON_BREV_SEL, PERON_BREV_STR, PERON_LONG_SEL, PERON_LONG_STR, PLANTFLEX_SEL, PLANTFLEX_SPAS, PLANTFLEX_STR, POST_TIB_SEL, POST_TIB_SPAS, POST_TIB_STR, RECT_FEM_SPAS

## Gait Features

We consider the following kinematic curves: Pelvis (all planes), Hip (all planes), Knee (all planes), Ankle (sagittal), Foot (transverse)

We consider the following moment curves: Hip (sagittal, coronal), Knee (sagittal, coronal), Ankle (sagittal)

We consider the following power curves: Hip, Knee, Ankle

We extract values (and timing) at the following instants: initial contact, opposite foot off, mid-stance, opposite foot contact, foot off, and mid-swing

We compute the following statistics (and timing where applicable): mean stance, maximum in stance, minimum in stance, mean swing, maximum in swing, minimum in swing

We also include the gait deviation index (GDI) and walking dynamic motor control (DMC)
