# Supplementary figures and images for: Demonstrating the utility of Instrumented Gait Analysis in the treatment of children with cerebral palsy
Source: PLoS One. 2024 Apr 9;19(4):e0301230. doi: 10.1371/journal.pone.0301230 (PMC11003627; doi:10.1371/journal.pone.0301230)

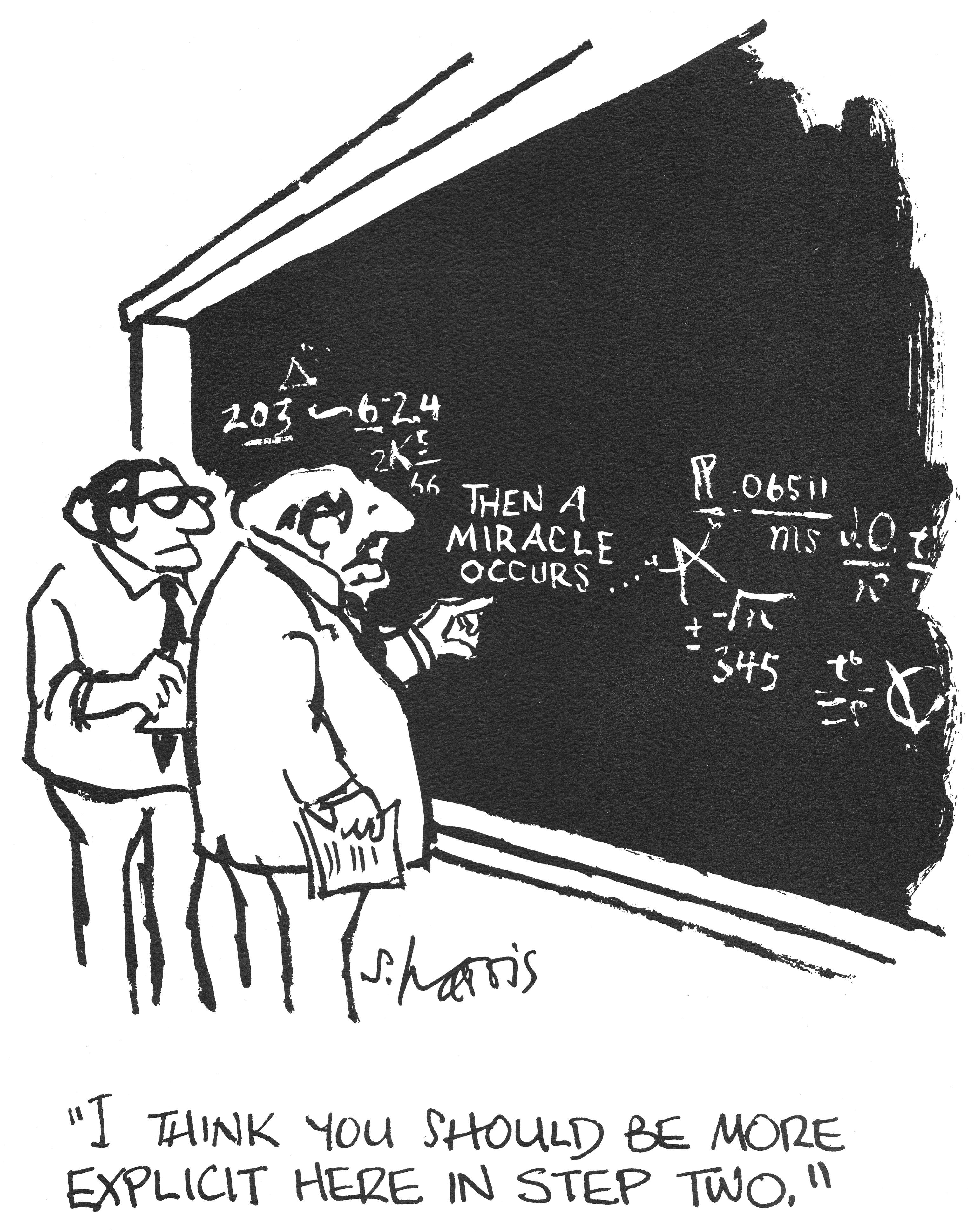

Supplement: S1 File — (ZIP) [file pone.0301230.s002.zip › Utility_Shareable/DATA/Then-a-Miracle-Occurs.jpg]
